# Environment and Scheduling Effects on Sprint and Middle Distance Running Performances

**DOI:** 10.1371/journal.pone.0079548

**Published:** 2013-11-20

**Authors:** Amal Haïda, Frédéric Dor, Marion Guillaume, Laurent Quinquis, Andy Marc, Laurie-Anne Marquet, Juliana Antero-Jacquemin, Claire Tourny-Chollet, François Desgorces, Geoffroy Berthelot, Jean-François Toussaint

**Affiliations:** 1 IRMES (biomedical Research Institute of Sports Epidemiology, Paris, France), INSEP (Institut National du Sport de l’Expertise et de la Performance), Paris, France; 2 Université de Rouen, Faculté des Sciences du Sport et de l’Education Physique CETAPS (Centre d’Etude Transformations des Activités Physiques et Sportives), Mont-Saint-Aignan, France; 3 Paris Descartes University, Sorbonne, Paris Cité, Paris, France; 4 Hôtel-Dieu Hospital, CIMS (Centre d’Investigations en Médecine du Sport), AP-HP (Assistance Publique-Hôpitaux de Paris), Paris, France; Universidad Europea de Madrid, Spain

## Abstract

**Purpose:**

Achievement of athletes’ performances is related to several factors including physiological, environmental and institutional cycles where physical characteristics are involved. The objective of this study is to analyse the performance achieved in professional sprint and middle-distance running events (100 m to 1500 m) depending on the organization of the annual calendar of track events and their environmental conditions.

**Methods:**

From 2002 to 2008, all performances of the Top 50 international athletes in the 100 m to 1500 m races (men and women) are collected. The historical series of world records and the 10 best annual performances in these events, amounted to a total of 26,544 performances, are also included in the study.

**Results:**

Two periods with a higher frequency of peak performances are observed. The first peak occurs around the 27.15^th^ ±0.21 week (first week of July) and the second peak around 34.75^th^ ±0.14 week (fourth week of August). The second peak tends to be the time of major international competitions (Olympic Games, World Championships, and European Championships) and could be characterized as an institutional moment. The first one, however, corresponds to an environmental optimum as measured by the narrowing of the temperature range at the highest performance around 23.25±3.26°C.

**Conclusions:**

This is the first study to demonstrate that there are two performance peaks at a specific time of year (27th and 34th weeks) in sprint and middle distance. Both institutional and ecophysiological aspects contribute to performance in the 100 m to 1500 m best performances and define the contours of human possibilities. Sport institutions may take this into account in order to provide ideal conditions to improve the next records.

## Introduction

Since the beginning of the modern Olympic era (1896), the best performance (BP) are in a process of exponential growth which now seems to have reached its limits [1]. Performance is often understood as a very broad term which involves many components such as : psychomotor abilities, flexibility and joint stiffness, muscle strength and power [2]. Athletes, like any living organism, depend on physiological regulations that respect the nycthemeral, seasonal or vital cycles [3]. There are variations in physiological factors such as maximum oxygen consumption (VO_2_ max) or concentrations of melatonin on the basis of seasonal rhythms [4]. This seasonal rhythmicity has been demonstrated for certain factors such as mood [5], lung function [6] and the core body temperature. It is also observed in the physical activity of the general population, which tends to be higher during summer [7]–[9]. Chan and colleagues (2006) [9] found a significant change in physical activity in the general population, as number of steps walked per day being related to temperature, precipitation and wind speed.

There is a limited amount of research that has investigated effects of seasonality in sports on sprint athletes’ performances. Yet the annual schedule of events seems to be a contributing factor to performance. Comparison of track and field world records (WR) shows that performance prevails in summertime. The influence of environmental parameters on physiology (ecophysiology) partly determines the evolution of human performance [10], [11]. Marathon optimal performances are set at a temperature around 10°. This performance dependency on temperature occurs not only for elite-standard athletes but for all participants also [12]–[14].

The objective of this study is to compare the date and temperature of the BP in sprint and middle distance races (100 m to 1500 m) for men and women during the annual calendar of international competitions, and observe their evolution over the Olympic era in order to assess the environment and scheduling effects on sprint and middle distance running performances.

## Methods

### Data Collection

From 2002 to 2008, all performances of the Top 50 international athletes in running events ranging from 100 m to 1500 m races for men and women were collected from the official website of the International Association of Athletics Federations (IAAF) [15]. For each event, data collection includes: full name of the athlete, the completion date and place of the competition: 23,746 performances are collected, 11,813 from males and 11,933 from females. Performances are divided into five categories defining the performance as a percentage in relation to the BP obtained at the event. The percent categories (PC) used were: [95–96%], [96–97%[, [97–98%[, [98–99%], [99–100%]. Within each group, performances were collected according to the competition type: (i) major competitions (the Olympic Games (OG), World Championships (WC), European Championships (EC) and American selections (US)), (ii) the international circuit represented by the Golden League and (iii) other meetings.

Date, place and name of the athlete when WR were set for the same distances between 1952 and 2010 are collected (181 WR) and the performances of the top 10 male and female 100 m race are gathered from 1891 to 2008, representing 2,617 performances.

Temperatures for each city, at the time of the competition, are recorded from 97 to 100 PC in 100 m, 200 m, 400 m, 800 m and 1500 m. In order to improve resolution, half PC are defined to study temperature density: [97–97.5%], [97.5–98%], [98–98.5%], [98.5–99%], [99–99.5%], [99.5–100%]. Temperature data are collected from the weather underground website [16].

The total number of performances collected for this study is 26,544.

### Statistical Analysis

#### Distribution of performance by PC

**Figure 1:**
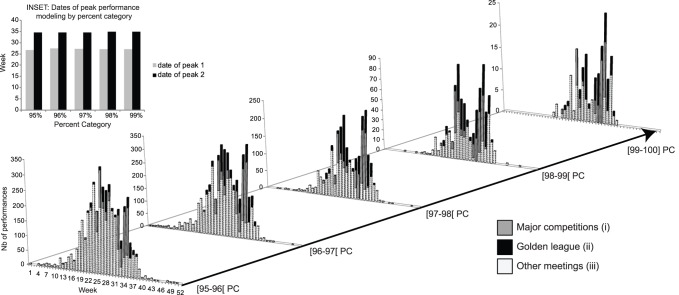
Figure 1. Number of performances per week and per percent category (PC) by (i) major competitions (Olympic Games (OG), World Championships (WC), American selections (US), European championships (EC)), (ii) Golden League and the others meetings (iii). INSET: Dates (week) of peaks performance modeling by PC.

The performance data from 2002 to 2008 is based on the distribution of performance per week of the year depending on the PC and the type of competition: mathematical analysis and modeling are done using Matlab. To estimate the two dates when the greatest numbers of performances occur, two functions are adjusted using the least squares method: the double Gaussian and double Lorentzian functions. For each PC, the best-fitted function is selected on the basis of adjusted R^2^ and the mean square error (RMSE) (See Materials and Methods S1, Figure S1, Tables S1 and S2). The two dates of peak performance are estimated using the elected model for each PC (Inert Figure 1). The proportion of performances in the two peaks is estimated by computing the area under the curve (proportion of performances) of each elected model and for each PC (See Materials and Methods S1, Figure S2, Table S2).

### Distribution of WR

Effect size for One-Way ANOVA is Cohen’s *d* and is evaluated with Cohen’s conventional criteria [17]. It is used to study the stability of the WR mean date by decades (1952–1959, 1960–1969, 1970–1979, 1980–1989, 1990–1999, 2000–2010). Statistical significance is considered at p<0.05.

### Temperature

For the temperature, the statistical analyses are done on R, Version 3.0.0 (R Core Team, Vienna, Austria, 2013) and results are expressed as a mean ± standard deviation. Fisher test is used to compare the dispersions between the different PC with a value of p<0.05 considered significant.

We estimate the density of temperature degrees for each of the PC over a homogeneous mesh of 5*6 nodes. The resolution used is of 7.5°C in the x-axis (temperature) and 0.59 percent in the y-axis PC.

## Results

### Distribution of Performance by PC and Competition

The distribution of weekly performances for each PC (95 to 100 PC) over the competition calendar shows two high frequency periods (Figure 1). The estimated dates of the two peak performances are constant within all PC (on mean 27.15^th^ ±0.21 week for peak 1 and 34.75^th^ ±0.14 for peak 2) (Figure 1, Inset).

Reaching the highest level, the areas under the curves of both peaks converge toward the same 50% value (See Figure S2).

The number of performances during major competitions (OG/WC/US/EC) increases from 16.7% for the 95 PC to 25.7% for the 99 PC. The performances recorded during the Golden League increase from 7.7% for the 95 PC to 29.1% for the 99 PC. Conversely, the number of performances in the other competitions decreases from 75.6% to 45.1% (Figure 1) (See Table S3).

### Distribution of WR

**Figure 2:**
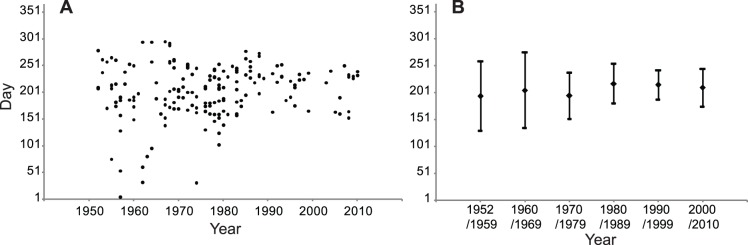
A. Distribution of world records (WR) date (day) in 100 m, 200 m, 400 m, 800 m and 1500 m running events from 1952 to 2010. B. Mean distribution of world records (WR) date (day) in 100 m, 200 m, 400 m, 800 m and 1500 m running events by decade from 1952 to 2010. The mean date is: 206,09 th day.

The mean distribution of WR date by decade from 1952 to 2010 is concentrated at the 206.09^th^ day ±46.17. The variability of WR date decreases considerably. In the first period (1952–1959), SD is 64.34, in the last period (2000–2010), SD is 35.09. However the mean day remains stable throughout the period (p = 0.29) (Figure 2), with a large effect size (*d* = 0.98).

### Influence of Temperature on Performance

**Figure 3:**
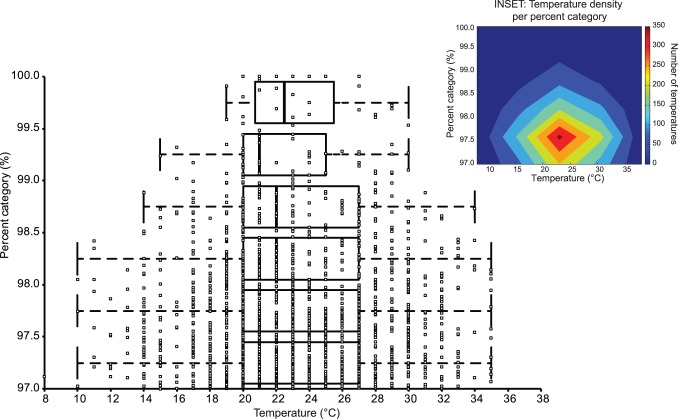
Percent category (PC) depending on temperature: comparison of temperature at different level from 97 PC to 100 PC in 100 m, 200 m, 400 m, 800 m and 1500 m. Respectively, in each half PC the mean are 23.12°C, 23.49°C, 23.23°C, 22.89°C, 22.63°C and 23.25°C and the median are 23.00°C, 23.00°C, 22.00°C, 22.00°C, 21.00°C and 22.50°C. INSET: Temperature density (ie. number of recorded temperatures) per PC computed over a mesh. The maximal density is computed at 23°C and 97.59% and progressively decreases as PC increase (due to the decrease in performance number). The density decreases as temperature increases or decreases from the maximal density (due to the effect of temperature on performance).

The analysis of the distribution of PC according to temperature shows a restriction in the thermal interval when reaching the highest performance level. This interval narrows from 10–32°C at 97 PC of the BP to 20–27°C for the 100 PC with a mean temperature of 23.23±4.75°C. Subdividing the data into PC, the mean temperature is 23.13±4.80°C for the PC [97 to 97.5[, 23.49±4.88°C for the PC [97.5 to 98[, 23.23±4.92°C for the PC [98 to 98.5[, 22.89±4.56°C for the PC [98.5 to 99[, 22.63±3.72°C for the PC [99 to 99.5[and 23.25±3.26°C for the BP [99.5 to 100]. Figure 3 highlights the narrowing of the temperature range at the BP interval. The peak value of the density mesh is 362 temperature values at 23°C and at 97.59%. The density decreases in both dimensions (temperature and PC) from this point confirming the mean temperature value stated above (Inset, Figure 3).

### Top 10 Sprinters from 1891 to 2008

**Figure 4:**
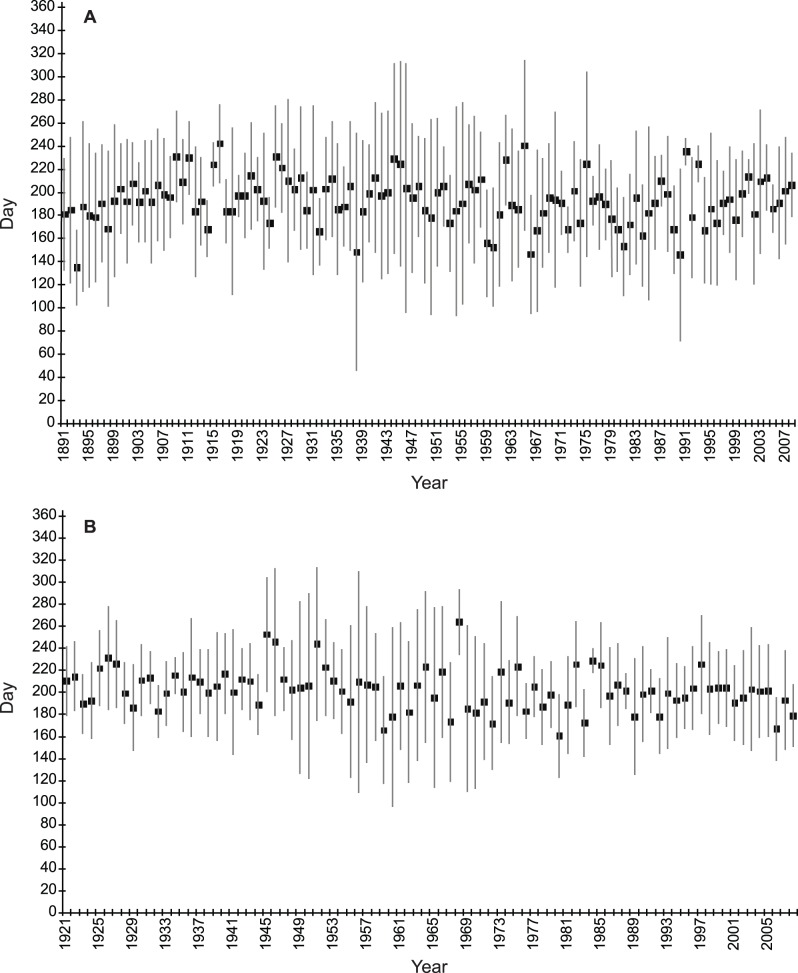
Relation between day of the performance and year in men and women. **A.** Average day of the achievement of the performance in the top 10 at the 100^th^ ±49.77. **B.** Average day of the achievement of the performance in the top 10 at the 100 m women since 1921. For all years combined, the average day is the 202.52^th^±44.0.

There is no evolutionary trend in the completion date on the 100 m performances throughout the modern Olympic era. Since 1891, men accomplish their best performance around July 10^th^ (±50 days) *id est* during the 28^th^ week and since 1921, women perform best around July 20^th^ (±44 days) *id est* during the 29^th^ week (Figure 4).

## Discussion

Our study is the first to our knowledge to analyze the exhaustiveness of the best performers in sprint and middle distance races in relation to temperature.

Previous studies have mostly analysed seasonality in the rhythms of daily life [2], [18] or in marathon runners [12] but no studies have demonstrated effects of seasonality through environmental or institutional conditions on performance in sprint.

Two yearly performance peaks are observed for all levels in this study. The first peak corresponds to the 27^th^ week of the year (first week of July) suggesting an environmental optimum for sprint events. The second peak occurs at the 34^th^ week (fourth week of August), which is related to the main sporting events such as: Olympic Games, European and World Championships. As seen in Figure 1, both peak dates are stable throughout all performance categories.

### Cultural Peak at the 34^th^ Week

The impact of major international competitions corresponds to the performance peak in August. The calendar scheduling for world championships or Olympic Games can be considered as an institutional attractor. IAAF hosts competitions taking place outdoor between February and October. Major competitions such as the World Championships, European championships and Olympic Games are usually scheduled in August whereas the international circuit of the Golden League covers the whole period between June and September. National federations plan their own schedules proposing competitions that allow their athletes to qualify for the major competitions.

This study highlights the existence of a cultural peak (second peak) occurring at the same times as the major international events. Globally, top athletes focus on the same goal: to be the most physically and mentally fit for this time of the year (Figure 1). This second peak corresponds to the athlete’s own planning for major competitions, which is a result of long term training, technical analysis, strategic choice, awareness of physical and psychological limits [19]–[21].

Although, training and preparation are essential to reach a BP at a specific moment, environmental factors will allow the achievement of the highest level of performance.

### Thermal Peak at the 27^th^ Week

The analysis of WR (Figure 2) and the top 10 BP in 100 m sprint (men and women) illustrates this first peak (Figure 4). Numerous studies have demonstrated effects of environmental conditions on the performance of marathon runners [22], [23].

Marathon requires a number of appropriate environmental conditions for thermoregulation of any runner, elite or amateur. The humidity, barometric pressure, dew point, and temperature are all essential in the quest of achieving optimal performance [24].

A recent study analysed the impact of environmental parameters on the performance of marathon running. It established a distribution of performances depending on temperature, observed regardless of the athlete’s level. This distribution function defines the field limits of the human possibilities [12]. The impact of temperature and season on biological parameters is largely documented in the literature [24]–[26].

In this present study, the results show a distribution for top performance in sprint and middle-sprint where the effective temperature range decreases with performance level (10–32°C at the bottom (97 PC of the BP); 20–27°C at the top (100 PC of the BP)) (Figure 3). Competitions are mainly organized in the northern hemisphere. The range of temperatures collected from the different host cities was large: ranging from 10 to 38°C but the mean temperature when achieving the BP is 23.23±4.75°C.

The standard deviations decrease progressively with increasing level, but all categories remain centered on the 23.23°C value. This suggests a very regulated process at all performance levels.

#### The effects of temperature on biological parameters

All biological structures and processes (human or not) are affected by temperature in thermodynamical regulations [27]. Performance depends on physiological responses to exercise performance in an interaction between body temperature and environmental temperature [26]. Performance decreases progressively as the environmental heat stress increases [25]. As with other biological rate processes, muscle function is strongly influenced by temperature. Specifically, muscle contraction rates (the rates of both force development and relaxation) are accelerated by an increase in temperature in both invertebrates and vertebrates [28], [29]. Fundamental biological functions like metabolic activity synchronize with the rhythmic phases of environmental change such as temperature. For gradually intensity increasing aerobic exercise the plasma concentration of certain ions (K^+^, Ca^2+^) and lactic acids appear differently when muscular exercise takes place at thermal neutrality (21°C) in comparison to exercise performed at 0°C [30].

At the favorable season, body temperature and metabolic rates increase and so does growth rate. Mammal growth depends on seasonal variation even for their bones structure [31]. Climates and seasons have a marked influence on human biology [18] including mental abilities [32], sexual activity [33] or territorial conflicts [34].

#### Temperature and mortality

Many chronobiological health aspects depend on season and temperature cycles. Affective disorders show a predictable onset in the fall/winter months and, reversely, a reduction in the spring/summer period [32]. Large-scale population studies have shown seasonal variations in mortality rates in different parts of the world peaking during the cold winter months [35], [36]. Relations between mortality and cardiovascular disease (CVD) in the winter months have been reported for many countries and might be partly explained by seasonal changes of risk factors. Cardiac death also depend on the season even after adjustment for age, cholesterol, blood pressure, and body mass index [36], [37]. Several studies have reported the existence of optimal ranges of air temperatures [38], [39]. Specifically, cold weather has been reported to be associated with increased risk of death from cardiovascular causes and respiratory infections [39]–[41]. The mortality rate is lower on days in which the maximum temperatures range between 20–25°C [38]. This means that survival rate is highest at this temperature range.

Our results show a mean temperature of 23.23±4.75°C for the BP which is converging with the temperature of the lowest mortality rates. Therefore, both survival capability and physiological capacities of the human are optimal at 20–25°C.

The two peaks of performance change its distribution in function of the performance level (See Figures S1 and S2). However, the first peak which corresponds to a “thermal peak” persists even at the highest level of competition. This demonstrates that despite the presence of an institutional attractor, represented by the major competitions, the environmental attractor remains omnipresent with an ideal temperature period for maximal performance. The adequacy of the thermal peak is as important as the cultural peak at the highest level.

## Conclusion

The range of possible combinations of environmental and institutional components is narrowed for the top performers. For sprint and middle-sprint races, when progressing toward the highest levels of performance, the importance of the institutional component regularly increases with a balanced effect for the top performers.

The novelty of this study is that, environmental conditions must be taken into account in order to achieve maximal speed. This field of possibilities reveals an ideal temperature to achieve optimal performance.

Calendars for major competitions should take this into account in order to increase the probability of breaking the next records.

## Supporting Information

Figure S1 **Date of peak performance modeling for 95% to 99% categories.** The Double Lorentzian (continuous line) and Double Gaussian functions (broken line) are adjusted to each percent category. Although the two models differ in the estimate of the tails, they roughly provide the same estimates of x_01_ and x_02_ (maximum difference is around 0.5 week).
(EPS)

Figure S2 **Area under the curve (AUC) for the elected functions and both peaks in each PC.** Equations (10) and (11) are used to estimate p_1_ and p_2_. The AUC of both peaks converge toward a unique value as the PC increase (50%). **The proportion of performances:** The estimation of the area under the curve for the two peaks shows that, when increasing the PC, the proportion of performances in each peak progressively converge to the same value, from 93.67% (peak 1) vs. 6.33% (peak 2) for PC = 95% to 50.65% (peak1) vs. 49.35% (peak2) with PC = 99% (Figure S2).
(EPS)

Table S1 **Statistics of the two models.** For each percent category, the adjusted R^2^, rMSE and sse are given. Statistics of the elected function are mentioned in bold.
(DOC)

Table S2 **Results of the two models.** For percent category and model, the estimated date of peak (x_01_, x_02_), value of peak (f(x_01_), f(x_02_)), the total proportion of performance (area under the curve), and p_1_, p_2_ are given. Results of elected function are given in bold.
(DOC)

Table S3 **Number of performances per depending on the type of competition and the percent category.** N is the number for different percent category (PC) and its equivalent percentage. On the overall performance analyzed, 2,347 were conducted during major competitions and 2,093 during the Golden League. The other 8,079 performances were done in other competitions (OG: Olympic Games; WC: World championships; US: American selections; EC: European Championships).
(DOC)

Materials and Methods S1 **The two models (double Gaussian and double Lorentzian functions) are presented.** The methods for estimating the dates of the two peaks and the area under the curve are described.
(PDF)
