# Supplementary material for: Environment and Scheduling Effects on Sprint and Middle Distance Running Performances
Source: PLoS One. 2013 Nov 20;8(11):e79548. doi: 10.1371/journal.pone.0079548 (PMC3868388; doi:10.1371/journal.pone.0079548)
Supplement: Table S1 — Statistics of the two models. For each percent category, the adjusted R2, rMSE and sse are given. Statistics of the elected function are mentioned in bold. (DOC) [file pone.0079548.s003.doc]

|  |  | **Double Gaussian (DG)** | **Double Lorenz (DL)** |
| --- | --- | --- | --- |
| 95 PC | **R² ajusted** | **0,9516** | 0,9305 |
|  | **rMSE** | **22,7891** | 27,3043 |
|  | **sse** | **23889,8006** | 34294,2 |
| 96 PC | **R² ajusted** | 0,9291 | **0,9296** |
|  | **rMSE** | 29,5593 | **29,439** |
|  | **sse** | 40192,7026 | **39866** |
| 97 PC | **R² ajusted** | 0,9 | **0,9333** |
|  | **rMSE** | 21,6801 | **17,7025** |
|  | **sse** | 21621,279 | **14415,4** |
| 98 PC | **R² ajusted** | 0,893 | **0,9133** |
|  | **rMSE** | 8,1656 | **7,348** |
|  | **sse** | 3067,1187 | **2483,7** |
| 99 PC | **R² ajusted** | **0,832** | 0,8242 |
|  | **rMSE** | **2,3597** | 2,4138 |
|  | **sse** | **256,141** | 268 |

**Table S1. Statistics of the two models.** For each percent category (PC), the adjusted R², rMSE and sse are given. Statistics of the elected function are mentioned in bold.
