# Supplementary material for: Environment and Scheduling Effects on Sprint and Middle Distance Running Performances
Source: PLoS One. 2013 Nov 20;8(11):e79548. doi: 10.1371/journal.pone.0079548 (PMC3868388; doi:10.1371/journal.pone.0079548)
Supplement: Table S2 — Results of the two models. For percent category and model, the estimated date of peak (x01, x02), value of peak (f(x01), f(x02)), the total proportion of performance (area under the curve), and p1, p2 are given. Results of elected function are given in bold. (DOC) [file pone.0079548.s004.doc]

| **95 PC** |  |  |
| --- | --- | --- |
| **Estimates** | **DG** | **DL** |
| x_01_ (date) | **26,8351** | 26,5746 |
| x_02_ (date) | **34,6752** | 34,5948 |
| f(x_01_) | **280,7254** | 309,4273 |
| f(x_02_) | **276,1141** | 327,4228 |
| Area under the curve | **4302,7845** | 4737,4484 |
| P_1_ (%) | **93,6664** | 90,3258 |
| P_2_ (%) | **6,3336** | 9,6742 |
| **96 PC** |  |  |
| x_01_ (date) | 27,8848 | **27,3995** |
| x_02_ (date) | 34,6783 | **34,6435** |
| f(x_01_) | 280,9383 | **321,5128** |
| f(x_02_) | 369,4804 | **385,7318** |
| Area under the curve | 4430,1672 | **4738,1772** |
| P_1_ (%) | 90,8454 | **82,7868** |
| P_2_ (%) | 9,1546 | **17,2132** |
| **97 PC** |  |  |
| x_01_ (date) | 27,0853 | **27,2493** |
| x_02_ (date) | 34,7478 | **34,6277** |
| f(x_01_) | 172,4108 | **201,6900** |
| f(x_02_) | 225,5263 | **259,8931** |
| Area under the curve | 2332,2075 | **2551,2424** |
| P_1_ (%) | 65,7555 | **64,5186** |
| P_2_ (%) | 34,2445 | **35,4814** |
| **98 PC** |  |  |
| x_01_ (date) | 27,2077 | **27,0652** |
| x_02_ (date) | 35,0370 | **34,8905** |
| f(x_01_) | 71,9553 | **83,8032** |
| f(x_02_) | 77,6709 | **84,8193** |
| Area under the curve | 778,2088 | **858,3401** |
| P_1_ (%) | 51,4414 | **50,4806** |
| P_2_ (%) | 48,5586 | **49,5194** |
| **99 PC** |  |  |
| **Estimates** | **DG** | **DL** |
| x_01_ (date) | **27,2122** | 27,3908 |
| x_02_ (date) | **34,8983** | 34,8036 |
| f(x_01_) | **11,4049** | 12,1392 |
| f(x_02_) | **19,9517** | 23,2029 |
| Area under the curve | **172,2617** | 188,3966 |
| p_1_ (%) | **50,6497** | 51,9671 |

**Table S2. Results of the two models.** For percent category and model, the estimated date of peak (x_01_, x_02_), value of peak (f(x_01_), f(x_02_)), the total proportion of performance (area under the curve) , and p_1_, p_2_ are given. Results of elected function are given in bold.
