# Supplementary material for: Environment and Scheduling Effects on Sprint and Middle Distance Running Performances
Source: PLoS One. 2013 Nov 20;8(11):e79548. doi: 10.1371/journal.pone.0079548 (PMC3868388; doi:10.1371/journal.pone.0079548)
Supplement: Table S3 — Number of performances per depending on the type of competition and the percent category. N is the number for different percent category (PC) and its equivalent percentage. On the overall performance analyzed, 2,347 were conducted during major competitions and 2,093 during the Golden League. The other 8,079 performances were done in other competitions (OG: Olympic Games; WC: World championships; US: American selections; EC: European Championships). (DOC) [file pone.0079548.s005.doc]

|  | Percent category | 95% | | 96% | | 97% | | 98% | | 99% | |
| --- | --- | --- | --- | --- | --- | --- | --- | --- | --- | --- | --- |
| Type of competition |  | n | % | n | % | n | % | n | % | n | % |
| Major competitions | OG, WC, US, EC | 712 | *16.7* | 826 | *18.4* | 576 | *23.0* | 188 | *22.4* | 45 | *25.7* |
|  | Golden League | 461 | *7.7* | 743 | *16.5* | 591 | *23.6* | 247 | *29.4* | 51 | *29.1* |
| Others meetings |  | 3309 | *75.6* | 2930 | *65.1* | 1336 | *53.4* | 405 | *48.2* | 79 | *45.1* |

**Table S3. Number of performances per depending on the type of competition and the percent category.** N is the number for different percent category and its equivalent percentage. On the overall performance analyzed, 2,347 were conducted during major competitions and 2,093 during the Golden League. The other 8,079 performances were done in other competitions (OG: Olympic Games; WC: World championships; US: American selections; EC: European Championships)
