# Supplementary material for: Environment and Scheduling Effects on Sprint and Middle Distance Running Performances
Source: PLoS One. 2013 Nov 20;8(11):e79548. doi: 10.1371/journal.pone.0079548 (PMC3868388; doi:10.1371/journal.pone.0079548)
Supplement: Materials and Methods S1 — The two models (double Gaussian and double Lorentzian functions) are presented. The methods for estimating the dates of the two peaks and the area under the curve are described. (PDF) [file pone.0079548.s006.pdf]

Supporting Materials and Methods

1) The double Gaussian and double Lorentzian
In order to estimate the two dates where the highest number of performances occurs for both thermal and cultural peak, we investigated two functions:

(i) The double Gaussian function $f_1(x)$ that is the sum of two Gaussian functions:

$$f_1(x) = f_{1,1}(x) + f_{1,2}(x) \tag{1}$$

where

$$f_1(x) = a_1 \cdot \exp^{-\left(\frac{x-b_1}{c_1}\right)^2} + a_2 \cdot \exp^{-\left(\frac{x-b_2}{c_2}\right)^2} \tag{2}$$

(ii) The double Lorentz function $f_2(x)$ that is the sum of two Lorentzian functions:

$$f_1(x) = f_{2,1}(x) + f_{2,2}(x) \tag{3}$$

where

$$f_2(x) = c_1 \frac{\frac{2}{a_1 \pi}}{1 + \left(\frac{x-b_1}{a_1/2}\right)^2} + c_2 \frac{\frac{2}{a_2 \pi}}{1 + \left(\frac{x-b_2}{a_2/2}\right)^2} \tag{4}$$

The functions f_1 and f_2 are two-peak functions and x is the number of elite performances in a week. Resulting adjusted R^2 and RMSE were gathered for both functions and the elected function for a given percent category was the function that presented the best statistics.

2) Estimates of x_{01}, x_{02}, p_1, p_2
For each elected function in each percent category, the two peaks x_{01}, x_{02} were estimated, and the proportions p_1, p_2 were given by estimating the area under the curve in the interval [0, 52] for the functions $f_{1,1}$, $f_{1,2}$, $f_{2,1}$, $f_{2,2}$. For notation convenience, we denoted i and j as the indexes of the functions, such as when $i = 1$ and $j = 1$, $f_{i,j}$ referred to $f_{1,1}$. Integration of the functions was given by:

$$\int_0^{52} f_{i,j}(x) = F_{i,j}(52) - F_{i,j}(0) \tag{5}$$

where

$$F_{1,j}(x) = -\sqrt{\pi} \times a_j \times c_j \times \operatorname{erf}\left(\frac{b_j - x}{c_j}\right) \tag{6}$$

and

$$F_{2,j}(x) = -2 \frac{c_j \times \tan^{-1}\left(\frac{2\,(b_j - x)}{a_j}\right)}{\pi} \tag{7}$$

where erf(x) is the error function and $tan^{-1}(x)$ the inverse tangent function. The proportion of performances in the two peaks was estimated by computing the area under the curve (proportion of Performances) of each elected model and for each PC:

$$\int_0^{52} f_1(x) = \int_0^{52} f_{1,1}(x) + \int_0^{52} f_{1,2}(x) \tag{8}$$

$$\int_0^{52} f_2(x) = \int_0^{52} f_{2,1}(x) + \int_0^{52} f_{2,2}(x) \tag{9}$$

And the proportions were calculated in percentages using:

$$p_1 = \frac{\int_0^{52} f_{i,1}(x)}{\int_0^{52} f_i(x)} \times 100 \tag{10}$$

$$p_2 = \frac{\int_0^{52} f_{i,2}(x)}{\int_0^{52} f_i(x)} \times 100 \tag{11}$$

Where $i = 1$ or 2, depending on the elected function $f_1(x)$ or $f_2(x)$ at each PC.
